# Evaluation of Possible Genotoxic Activity of Dirithromycin in Cultured Human Lymphocytes

**DOI:** 10.1155/2015/535490

**Published:** 2015-10-21

**Authors:** Ahmet Kayraldız, Lale Dönbak, Ayşe Yavuz Kocaman, Esra Köker, Şule Gökçe

**Affiliations:** ^1^Department of Biology, Science and Arts Faculty, Kahramanmaraş Sütçü İmam University, Kahramanmaraş, Turkey; ^2^Department of Biology, Science and Arts Faculty, Mustafa Kemal University, Antakya, Turkey

## Abstract

Dirithromycin antibiotic is a 14-membered lactone ring macrolide and is widely used in medicine to treat many different types of bacterial infections. In the present study, the possible genotoxicity of dirithromycin was evaluated in cultured human lymphocytes by using sister chromatid exchanges (SCEs), chromosome aberration (CA), and micronucleus (MN) tests and also cell proliferation kinetics such as mitotic index (MI), replication index (RI), and nuclear division index (NDI) were analyzed for cytotoxicity. Cell cultures were treated with four different concentrations of dirithromycin (37.75, 67.50, 125, and 250 *µ*g/mL) for 24 and 48 h periods. Dirithromycin significantly induced SCE and MN frequency at all concentrations in both 24 and 48 h treated cells. In addition, CA level has been markedly increased in the cells treated with almost all concentrations of dirithromycin for 24 (except 37.75 *µ*g/mL) and 48 h treatment periods as compared to control. However, MI, RI, and NDI values were not affected by the dirithromycin treatment (*p* > 0.05). The results of this study indicated that dirithromycin treatment caused genetic damage by increasing the level of cytogenetic endpoints, suggesting its genotoxic and mutagenic action on human lymphocytes *in vitro*.

## 1. Introduction

Dirithromycin is a macrolide glycopeptide antibiotic and is used for the treatment of the mild-to-moderate infections, acute bacterial exacerbations of chronic bronchitis, community-acquired pneumonia, pharyngitis/tonsillitis, and uncomplicated skin, as well as skin structure infections. It is a prodrug and is developed for oral administration [1, 2].

Dirithromycin is converted by nonenzymatic hydrolysis during absorption to microbiologically active metabolite 9-(S)-erythromycylamine. Erythromycylamine exerts its activity by binding to the 50S subunits of the 70S bacterial ribosome resulting in inhibition of protein synthesis and also formation of ribosomal subunits, 50S and 30S. Sixty to 90% of the administered dose is hydrolysed to erythromycylamine within 35 minutes after dosing and conversion to erythromycin in serum is virtually completed after 1.5 hours. The primary route of elimination of erythromycin is faecal/hepatic [3–5].

Common side effects caused by dirithromycin are reported as vomiting, headache, nausea, diarrhea, and abdominal pain [1, 6]. On the other hand, safety and effectiveness of dirithromycin in children under 12 years of age have not been established. However, there are no adequate and well-controlled data in human pregnancy. Besides, lifetime studies in animals have not been performed with dirithromycin to evaluate carcinogenic potential. Although there was evidence about genotoxic effects of many antibiotic groups [7, 8], no information exists on the genotoxicity of dirithromycin.

Therefore, in the present study, possible genotoxic effects of dirithromycin on human lymphocytes were examined by using different cytogenetic indicator and endpoint tests, sister chromatid exchanges (SCEs), chromosome aberrations (CA), and micronucleus (MN). Cell growth kinetics such as mitotic index (MI), replication index (RI), and nuclear division index (NDI) were also analyzed for determining cytotoxic effect of the antibiotic.

## 2. Materials and Methods

### 2.1. Test Substance

**Figure 1:**
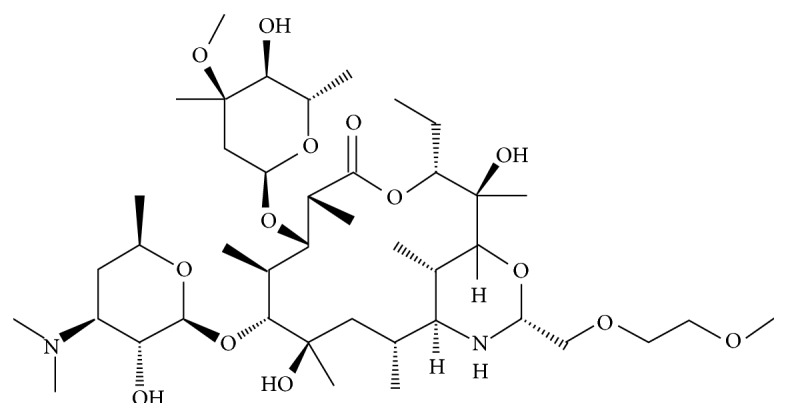
The chemical structure of dirithromycin.

Dirithromycin is a 14-membered lactone ring macrolide and is the C9-oxazine derivative of erythromycylamine, prepared by condensation, of the erthromycylamine with 2-(2-methoxyethoxy) acetaldehyde. The 9N, 11O-oxazine ring formed is a hemiaminal that is unstable under both acidic and alkaline aqueous conditions [1]. Chemical structure of dirithromycin is shown in Figure 1. Dirithromycin, trade name Dynabac, was purchased from Abdi İbrahim İlaç San. ve Tic. Aş. (İstanbul, Turkey) and dissolved in distilled water. Four doses, 37.75, 67.50, 125, and 250 *μ*g/mL, were prepared for the analysis as considered to be clinically relevant doses.

### 2.2. Collection of Blood Samples

Peripheral blood samples were taken from four healthy volunteer donors (two males and two females, aged 20–24 years) with no history of exposure to known mutagens. Approximately, 2 mL of venous blood was collected by venipuncture into injectors containing heparin as anticoagulant for the CA, SCE, and MN assays.

### 2.3. CA and SCE Assays

For duplicate peripheral lymphocyte cultures, 0.2 mL of heparinized whole blood samples was added to 2.5 mL chromosome medium B (Biochrom, F5023) supplemented with 10 *μ*g/mL bromodeoxyuridine (Sigma, B5002). Cultures were incubated in the dark at 37°C for 72 h. The cells were treated with 37.75, 67.50, 125, and 250 *μ*g/mL of dirithromycin for 24 h (dirithromycin was added 48 h after initiating the culture) and 48 h (dirithromycin was added 24 h after initiating the culture). A control and a known mutagen Mitomycin-C (Kyowa, 50-07-7) as positive control were also included. The cells were exposed to 0.06 *μ*g/mL colchicine (Sigma, C9754) for the last 2 h of culture. Centrifuged cells were harvested by 0.4% KCl as hypotonic solution and methanol : glacial acetic acid (3 : 1) as fixative. The staining of slides was carried out according to the standard methods using 5% Giemsa (Merck, 45380) for CA analysis [9] and modified fluorescence plus Giemsa method for SCE analysis [10].

Microscopic examination of the slides for CA and SCE was performed at 1000 magnification using oil immersion lens of Olympus binocular microscope. 100 well-spread metaphases per donor (a total of 400 metaphases per concentration) were examined for the occurrence of structural and/or numerical alterations. The number of CA per cell (CA/cell) and the mean frequency of abnormal cells (Ab.C%) per concentration and treatment period was calculated. For determining the number of SCEs, 25 well-spread second-division metaphases were analyzed. Obtained results were used to determine mean number of SCEs per cell (SCEs/cell). In addition, 100 cells from each donor were scored for determination of the replication index (RI) and 3000 cells per donor were analyzed for calculation of mitotic index (MI).

### 2.4. MN Assay

Whole heparinized blood (0.2 mL) was cultured in 2.5 mL of chromosome medium B and incubated in 37°C for 72 h. The cells were treated with 37.75, 67.50, 125, and 250 *μ*g/mL of dirithromycin for 24 and 48 h periods. Cytochalasin B (Sigma, C6762) was added to the cultures in a final concentration of 5.2 *μ*g/mL at 44 h of incubation. After the additional 24 h incubation at 37°C, cells were collected by centrifugation. Following the treatment with hypotonic solution at room temperature, cells were fixed in methanol : glacial acetic acid (3 : 1) solution three times. Air-dried slides were stained with 5% Giemsa solution [11].

Micronucleus frequency was determined by scoring a total of 1000 binucleated cells from each donor at 400 magnification according to the criteria for MN analysis described by Fenech [11]. In order to determine the cytotoxic effect, nuclear division index (NDI) was calculated per donor by classifying 500 cells with regard to the number of nuclei they contained.

### 2.5. Statistical Analysis

The obtained data were indicated as arithmetic mean (*x*) ± standard error (SE). Comparison of the difference between the average spontaneous and induced frequencies was performed using Student's *t*-test. SPSS for windows 10.0 package program was used for the statistical analysis. All *p* values were two-tailed and accepted significance level was < 0.05.

## 3. Results

The mean frequency of cytogenetic endpoints and the values of cell growth kinetics for each concentration of dirithromycin and controls are indicated in Tables 1–3. In this study, dirithromycin significantly induced SCE frequency at all concentrations in both 24 and 48 h treated cells (Table 1). The differences between the dirithromycin doses and control with regard to RI were not statistically significant (*p* > 0.05), as shown in Table 1.

**Figure 2:**
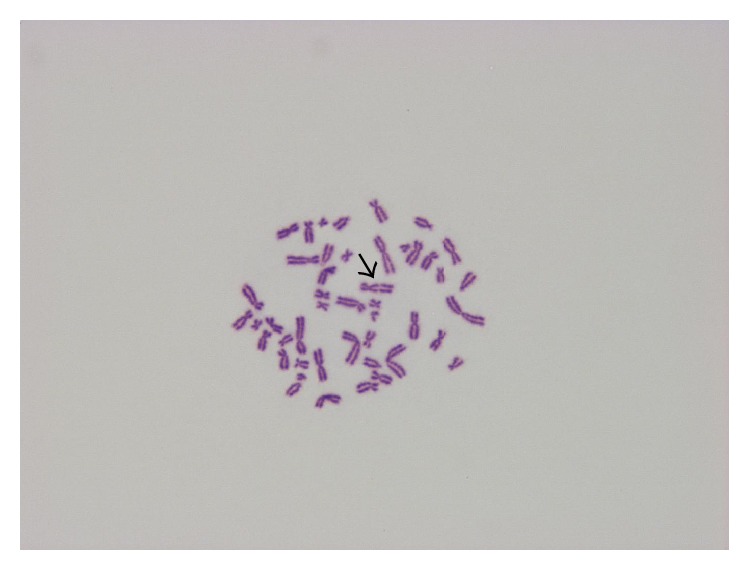
Chromosome break (67.50 *μ*g/mL dirithromycin, 24 h treatment, ♀).

**Figure 3:**
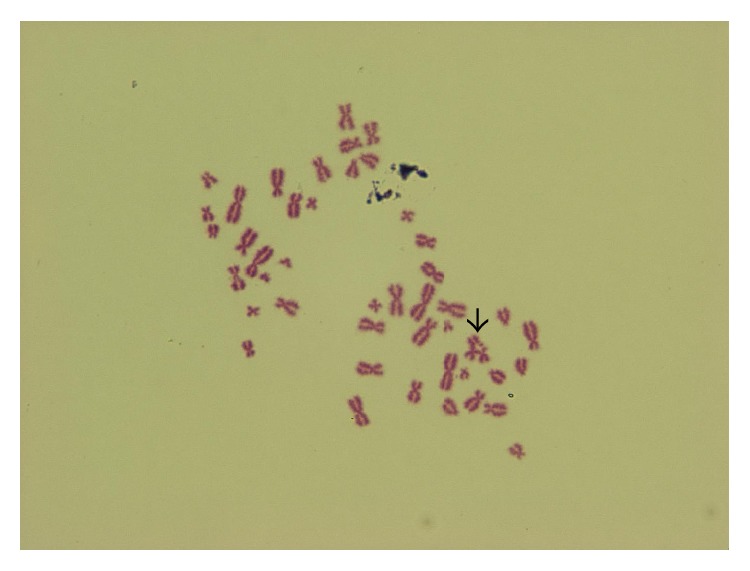
Chromatid exchange (250 *μ*g/mL dirithromycin, 48 h treatment, ♂).

**Figure 4:**
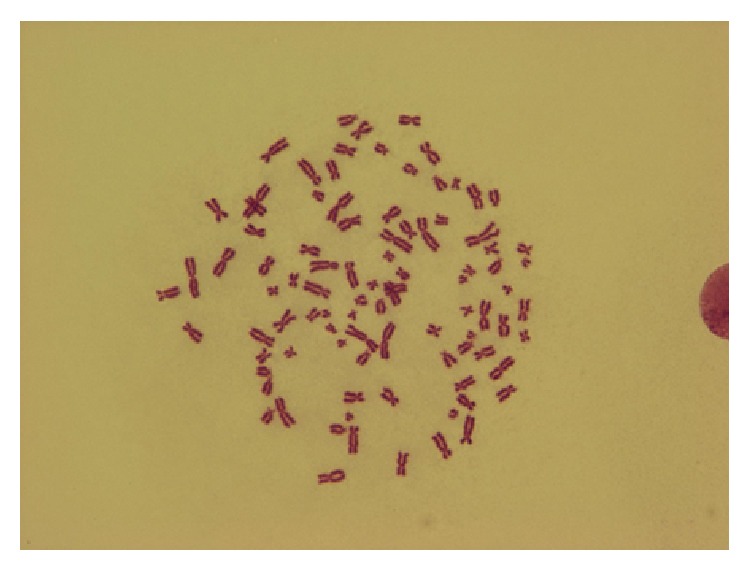
Polyploidy (125 *μ*g/mL dirithromycin, 24 h treatment, ♂).

Comparison of control and treated cells revealed that dirithromycin significantly increased the frequencies of CA and Ab.C at all concentrations for 24 (except lowest dose of 37.75 *μ*g/mL) and 48 h (Table 2). Six types of structural aberrations, namely, chromatid and chromosome breaks (Figure 2), chromatid exchange (Figure 3), fragment, sister union, and dicentric chromosome, and only one type of numerical aberration (polyploidy) (Figure 4) were observed. As shown in Table 2, dirithromycin treatment did not influence MI value significantly (*p* > 0.05).

All tested concentrations of dirithromycin caused a significant elevation in the MN level of cells for all treatment periods (Table 3). Analysis of the distribution of BNMN indicated that dirithromycin significantly enhanced the rate of binucleated cells containing MN, as compared to control. Table 3 also indicates that NDI values of cells were not affected by dirithromycin treatment (*p* > 0.05).

## 4. Discussion

The macrolides are bacteriostatic antibiotics with a broad spectrum of activity against many Gram-positive bacteria. Macrolide antibiotics include natural members, prodrugs, and semisynthetic derivatives and are composed of 14–16 member-ringed compounds. Although they are characterized by similar chemical structures and mechanism of action and resistance, they vary in the different pharmacokinetic parameters and spectrum of activity [2, 3].

Macrolides are protein synthesis inhibitors. The mechanism of action of macrolides is inhibition of bacterial protein biosynthesis. Macrolides exert their activity by reversibly binding to the 50S subunit of the 70S bacterial ribosome. They stimulate dissociation of peptidyl-tRNA molecule from the ribosome during the elongation phase. This results in chain termination. Thus RNA-dependent protein synthesis is suppressed and bacterial growth is inhibited. Macrolides tend to accumulate within leukocytes and are transported into the site of infection [2–4, 6].

It has been reported that some macrolides have toxic effects on the cardiovascular and gastrointestinal systems [12, 13] and that they also lead to allergy, liver injury, developmental toxicity, genotoxicity, and teratogenicity [14–18].

Since there was evidence about genotoxic effects of many antibiotic groups [7, 8] several researchers investigated the mutagenic and genotoxic potentials of macrolides. In the study of İla and Topaktaş [17], spiramycin (100 mg/kg bwt/day) induced the CA level in bone morrow cells of rats following oral treatment for 7 days. Spiramycin did not induce the level of CA and SCEs in the study of Rencüzoğulları et al. [19]. Acetyl-spiramycin, a derivative of spiramycin, was not mutagenic against* Salmonella* Typhimurium TA98, TA100, TA1535, TA1537, and TA1538 strains [20].

Another macrolide antibiotic, erythromycin, elevated the CA frequency in human lymphocytes [21] but it did not increase the MN frequency in treated human lymphocytes [22]. Grujičić et al. [23] reported that combined therapy with ritodrine, erythromycin, and verapamil over six days significantly increased the frequency of MN in peripheral blood lymphocytes of pregnant women. Tohamy [24] observed increased genotoxicity induced by drug-drug interaction between the antidepressant sertraline and the antibiotic erythromycin in bone-marrow cells of mice. Miokamycin, an antibiotic of macrolide group, was not mutagenic against* Salmonella* Typhimurium strains and also it did not induce dominant lethal mutations in mice [25]. İbrahim and El-Sherbeny [26] reported that clarithromycin induced chromosome aberrations in both bone-marrow and splenocyte cells of mice.

In the present study, we investigated the genotoxic potential of dirithromycin in human lymphocytes by using SCE, CA, and MN tests, since there is no information on its genotoxic potential. We also determined the cell proliferation kinetics, MI, RI, and NDI, values in the treated cells for analyzing the cytotoxic effect of the test substance. The concentrations of dirithromycin used in the experiments were determined based on daily adult dose taken by an individual (two tablets). Since a tablet contains 512.46 mg dirithromycin, an adult individual has to take 1024.96 mg dirithromycin in a day. After the calculations, we prepared the stock solution of 67.5 *μ*g/mL of dirithromycin with distilled water, which was relevant to daily dose of an adult. To identify the possible cases in the overdose and lower dose, we also prepared the twofold upper (125 and 250 *μ*g/mL) and onefold lower doses (37.75 *μ*g/mL). Then the human lymphocytes were treated with these four concentrations of dirithromycin for 24 and 48 h periods for determining an abnormality that occurred in first cell cycle (24 hours), whether repaired or not in the second cell cycle (48 hours).

Obtained results showed that all tested concentrations of dirithromycin caused a statistically significant increase in the frequencies of SCE and MN in human lymphocytes as compared to controls. Besides, dirithromycin markedly elevated the levels of CA and Ab.C at all concentrations for 24 and 48 h treatments with regard to controls. However, this increase was not statistically significant in the cells treated with the lowest dose, 37.75 *μ*g/mL, for 24 h treatment period (*p* > 0.05). On the other hand, dirithromycin did not influence the cell proliferation kinetics of MI, RI, and NDI values (*p* > 0.05). This indicated that dirithromycin has genotoxic activity in human lymphocytes but not cytotoxic effect.

## 5. Conclusion

Finally, on the basis of the results and assuming that SCEs, CA, and MNs can be used as cytogenetic biomarkers for chemical genotoxicity, it can be concluded that dirithromycin in the tested concentrations* in vitro* caused DNA damage and has genotoxic effects on human peripheral lymphocytes. Further studies should be performed on genotoxic potential of dirithromycin in different test system using clinically relevant doses.

## Figures and Tables

**Table 1:** Frequency of sister chromatid exchanges (SCEs/cell) and replication index (RI) in cultured human lymphocytes treated with dirithromycin and controls.

Test substance	Treatment		Min-max	SCEs/cell ± SE^a^	RI ± SE
	Time (h)	Cons. (*µ*g/mL)	SCE		
Control		0.0	1–7	4.89 ± 0.34	2.33 ± 0.11
Control (+)^b^		0.1	8–39	30.22 ± 1.60	1.76 ± 0.12
Dirithromycin	24	37.75	1–9	6.34 ± 0.33^*∗*^	2.15 ± 0.21
		67.50	1–9	6.54 ± 0.43^*∗*^	2.16 ± 0.13
		125.00	1–10	6.70 ± 0.35^*∗*^	1.93 ± 0.13
		250.00	2–12	7.92 ± 0.22^*∗∗*^	2.00 ± 0.10

Control		0.0	1–8	4.89 ± 0.34	2.33 ± 0.11
Control (+)		0.1	8–40	58.59 ± 0.88	1.57 ± 0.09
Dirithromycin	48	37.75	1–9	6.58 ± 0.32^*∗*^	2.28 ± 0.05
		67.50	1–11	7.28 ± 0.23^*∗∗*^	2.34 ± 0.06
		125.00	1–12	7.95 ± 0.26^*∗∗*^	2.30 ± 0.04
		250.00	1–13	8.02 ± 0.20^*∗∗*^	2.31 ± 0.04

^a^SE: standard error.

^b^Control (+): Mitomycin-C as positive control.

^*∗*^ *p* < 0.05; ^*∗∗*^ *p* < 0.01.

**Table 2:** Frequency of chromosomal abnormalities (CA/cell), abnormal cells (Ab.C), and mitotic index (MI) in cultured human lymphocytes treated with dirithromycin and controls.

Test substance	Treatment		CA/cell ± SE	Ab.C ± SE	MI ± SE
	Time (h)	Cons. (*µ*g/mL)		(%)	
Control		0.0	0.06 ± 0.00	6.75 ± 0.25	0.04 ± 0.01
Control (+)^b^		0.1	0.22 ± 0.03	21.50 ± 3.22	0.02 ± 0.00
Dirithromycin	24	37.75	0.09 ± 0.01	9.00 ± 1.22	0.05 ± 0.00
		67.50	0.14 ± 0.02^*∗*^	13.75 ± 1.88^*∗*^	0.04 ± 0.00
		125.00	0.17 ± 0.01^*∗∗*^	17.50 ± 1.32^*∗∗*^	0.04 ± 0.00
		250.00	0.21 ± 0.01^*∗∗*^	21.00 ± 1.22^*∗∗*^	0.04 ± 0.00

Control		0.0	0.06 ± 0.00	6.75 ± 0.25	0.03 ± 0.00
Control (+)		0.1	0.33 ± 0.04	32.50 ± 3.01	0.02 ± 0.00
Dirithromycin	48	37.75	0.16 ± 0.01^*∗∗*^	15.25 ± 1.49^*∗∗*^	0.05 ± 0.00
		67.50	0.17 ± 0.01^*∗∗*^	17.00 ± 1.29^*∗∗*^	0.07 ± 0.02
		125.00	0.20 ± 0.02^*∗∗*^	19.50 ± 1.50^*∗∗*^	0.07 ± 0.01
		250.00	0.28 ± 0.03^*∗∗*^	27.75 ± 4.15^*∗∗*^	0.06 ± 0.02

^b^Control (+): Mitomycin-C as positive control.

^*∗*^ *p* < 0.05; ^*∗∗*^ *p* < 0.01.

**Table 3:** Frequency of micronuclei (MNs), binucleated cells with MN (BNMN), and nuclear division index (NDI) in cultured human lymphocytes treated with dirithromycin and controls.

Test substance	Treatment		MN ± SE (‰)	BNMN ± SE (‰)	NDI ± SE
	Time (h)	Cons. (*µ*g/mL)			
Control		0.0	6.00 ± 0.40	6.00 ± 0.40	1.19 ± 0.02
Control (+)^b^		0.1	21.00 ± 1.77	18.00 ± 1.47	1.11 ± 0.01
Dirithromycin	24	37.75	9.25 ± 0.62^*∗∗*^	9.75 ± 1.10^*∗*^	1.17 ± 0.01
		67.50	11.25 ± 1.18^*∗∗*^	10.50 ± 0.95^*∗∗*^	1.16 ± 0.02
		125.00	13.25 ± 0.62^*∗∗∗*^	12.75 ± 0.47^*∗∗∗*^	1.16 ± 0.02
		250.00	18.50 ± 0.64^*∗∗∗*^	14.50 ± 0.64^*∗∗∗*^	1.14 ± 0.00

Control		0.0	6.00 ± 0.40	6.00 ± 0.40	1.19 ± 0.02
Control (+)		0.1	49.50 ± 3.12	46.00 ± 2.16	1.11 ± 0.00
Dirithromycin	48	37.75	14.00 ± 0.70^*∗∗*^	10.50 ± 0.50^*∗∗*^	1.25 ± 0.02
		67.50	15.25 ± 0.85^*∗∗∗*^	13.25 ± 0.47^*∗∗∗*^	1.23 ± 0.03
		125.00	14.50 ± 0.64^*∗∗∗*^	13.00 ± 0.40^*∗∗∗*^	1.42 ± 0.16
		250.00	15.50 ± 0.64^*∗∗∗*^	15.00 ± 0.70^*∗∗∗*^	1.25 ± 0.00

^b^Control (+): Mitomycin-C as positive control.

^*∗*^ *p* < 0.05; ^*∗∗*^ *p* < 0.01; ^*∗∗∗*^ *p* < 0.001.
